# Neurophysiological Markers of Reward Processing Can Inform Preclinical Neurorehabilitation Approaches for Cognitive Impairments Following Brain Injury

**DOI:** 10.3390/brainsci15050471

**Published:** 2025-04-29

**Authors:** Miranda Francoeur Koloski, Reyana Menon, Victoria Krasnyanskiy

**Affiliations:** 1 Mental Health Service, VA San Diego Healthcare System, La Jolla, CA 92161, USA; 2 Department of Psychiatry, UC San Diego, La Jolla, CA 92093, USA; 3 Center of Excellence for Stress and Mental Health, VA San Diego Healthcare System, La Jolla, CA 92161, USA

**Keywords:** neurophysiology, traumatic brain injury, reward processing, beta oscillations

## Abstract

Brain stimulation therapies may be used to correct motor, social, emotional, and cognitive consequences of traumatic brain injury (TBI). Neuromodulation applied with anatomical specificity can ameliorate desired symptoms while leaving functional circuits intact. Before applying precision medicine approaches, preclinical animal studies are needed to explore potential neurophysiological signatures that could be modulated with neurostimulation. This review discusses potential neural signatures of cognition, particularly reward processing, which is chronically impaired after brain injury. Electrophysiology, compared to other types of biomarkers, can detect deficits missed by structural measures, holds translational potential between humans and animals, and directly informs neuromodulatory treatments. Disturbances in oscillatory activity underscore structural, molecular, and behavioral impairments seen following TBI. For instance, cortico-striatal beta frequency activity (15–30 Hz) during reward processing represents subjective value and is chronically disturbed after frontal TBI in rodents. We use the example of evoked beta oscillations in the cortico-striatal network as a putative marker of reward processing that could be targeted with electrical stimulation to improve decision making after TBI. This review highlights the necessity of collecting electrophysiological data in preclinical models to understand the underlying mechanisms of cognitive behavioral deficits after TBI and to develop targeted stimulation treatments in humans.

## 1. Introduction

Biomarkers serve as indicators of biological processes that are objective and reproducible [1]. Categories of biomarkers include neuroimaging (magnetic resonance imaging (MRI)), neurophysiological (Electroencephalography (EEG)), biofluid (blood), and digital biomarkers (wearables), all of which are being implored to study traumatic brain injury (TBI). A number of TBI biomarkers have been cataloged, including serum-based protein, exosomal microRNAs, and metabolic indicators [2,3]. However, these molecular markers are limited in temporal precision and ability to reflect ongoing changes in brain activity, particularly in relation to cognition. Structural imaging (MRI) often fails to detect mild injuries. Although, new imaging advancements like diffusion tensor imaging have improved sensitivity to detect microstructural damage (axonal injury) [4,5]. To address these limitations, the impact of injury on neurophysiological activity (neural oscillations) can be examined. The search for biomarkers in TBI is extremely difficult given the heterogeneous nature of brain injury and the symptoms it produces. Even a single injury propagates multiscale disruptions to local and distributed brain areas. Since behavioral outcomes are hard to predict, establishing a reliable biomarker of injury that can estimate injury progression and response to treatment will be useful.

Brain injury triggers a cascade of insults to the central nervous system including a combination of inflammatory, cellular, and molecular changes [6,7,8]. Due to its superficial position in the brain, prefrontal cortex is particularly susceptible to injury [9,10,11]. Cortical damage leads to changes in reward-guided behavior, including impaired decision making, decreased motivation, impulsivity, and interrupted reinforcement-learning [11,12,13]. Most symptoms are resolved; however, in 5–15% of cases, cognitive deficits endure for years after injury [10,11]. Chronic symptoms are a result of cortical neuron loss, disconnection with networks, and neurotransmitter dysregulation [14,15,16].

The mechanism of injury determines if damage is focal (hematoma, hemorrhage, contusion), diffuse (axonal injury), or a combination. The most common type of injury is diffuse damage to the white matter tracts caused by shearing [17,18,19]. Diffuse axonal injury often fractures cortical-subcortical connectivity that can afflict cognitive networks and perpetuate neuropsychiatric symptoms [18,20]. The symptom profile is dependent on the neural pathways impacted. For example, thalamo-cortical tract damage accounts for significant alteration in executive dysfunction [21].

The goal of TBI treatment is to develop a precision medicine approach that targets a specific brain area or brain function (neurophysiological biomarker) that can be individualized based on each unique trauma pathology. Most treatment strategies center around addressing motor functions or physical impairments through rest and rehabilitation [22], leaving long-term cognitive deficits unaddressed. TBI-induced motor deficits, including gait, coordination, fine motor skills, seizures, and muscle tremors, can be alleviated with a combination of rehabilitation, brain stimulation, and pharmacologic strategies (muscle relaxation/motor inhibition drugs) [23,24,25]. There are no pharmacological treatments that specifically regulate TBI-related changes in mood and cognition or prevent the onset of neuropsychiatric disorders (which have a 2–3 times greater risk of developing) [11,16,26].

**Figure 1:**
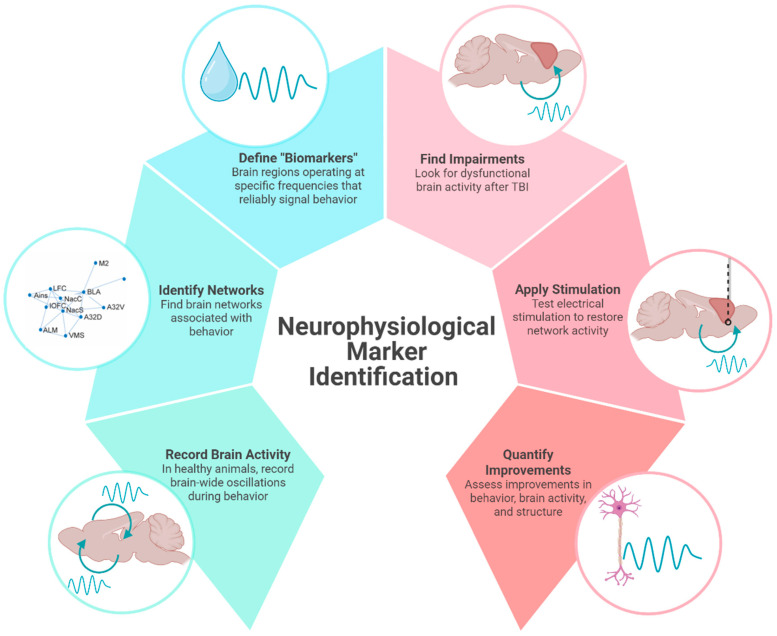
Neurophysiological marker identification. An illustrative overview of how identifying neural activity associated with distinct behaviors can be used to identify and test therapeutic targets in TBI. In this example of a preclinical study, brain activity recorded in healthy animals is used to identify brain networks associated with behavior. Neural signals with region and frequency specificity can be used to apply electrical stimulation to injured brains. In this review, we use beta frequency oscillations in the cortico-striatal network as a potential neurophysiological marker of reward processing that can be used to improve cognition in animals with TBI. Created in BioRender. Koloski, M. (2025) https://BioRender.com/7xr6lr3 (accessed on 21 April 2025).

In line with a precision medicine approach, using neuromodulation to provide electrical stimulation is thought to activate specific neural circuits, which can strengthen its functional network to improve desired behaviors. Identifying a neurophysiological biomarker associated with specific behaviors is needed to improve the precision and reliability of neuromodulation. As opposed to pharmacological interventions, neuromodulation has the potential to influence a multitude of brain areas while maintaining specificity for functional circuits. This review aims to synthesize findings across preclinical and clinical studies related to neurophysiological biomarkers in TBI, specifically oscillatory associated with reward-guided decision-making impairments seen chronically after injury. We provide a theoretical framework by which preclinical studies can support large-scale electrophysiological recordings in healthy animals to identify brain networks and potential “biomarkers” that can be targeted with electrical stimulation in injured animals (Figure 1). While not a formal meta-analysis, our narrative review offers converging evidence to support the translational potential of using electrophysiological signatures to identify new therapeutic targets for TBI.

## 2. Neurophysiological Markers Can Identify Potential Therapeutic Targets

### 2.1. Brain Oscillations and Their Disruption After Injury

Electrophysiological measures, including EEG, can detect neural changes after even a mild TBI that structural imaging may fail to detect [4,22]. For example, EEG can detect reduced amplitude and slowing of brain waves in the acute and subacute phases of mild TBI of patients without visible structural damage on MRI/CT scans [27]. The benefits of electrophysiological measures include good temporal resolution, translatability between humans and animals, affordability, usefulness during recovery, and the capacity to be paired with cognitive tasks [1,12]. Oscillatory activity in an EEG reflects coordinated neural activity that facilitates communication within and between brain areas. Oscillatory bands, which are operationally defined based on functional brain states, range from 0.5 to 500 Hz [28]. The average ionic movements (including synaptic activity, calcium fluctuations, spike after-hyperpolarization, gap junctions, glial, etc.) recorded from an electrode are known as the local field potential (LFP) [28]. LFP activity patterns are associated with distinct behaviors and can predict disease states or response to treatment [28,29].

Numerous studies have identified persistent abnormalities in oscillatory dynamics following TBI, including theta suppression during working memory tasks [30], delta elevations during wakefulness [31], and reductions in beta/gamma frequencies during cognition [32]. Generally, lower frequencies (8–30 Hz) are thought to reflect top–down information processing driven by thalamo-cortical connections and higher frequencies (40–100 Hz) to drive bottom–up processing [33,34]. Importantly, connectivity data, reflecting widespread changes across networks, better predict symptoms severity than local changes in power, suggesting brain injury should be treated as a network disorder (opposed to focal injury) [5]. Changes in oscillations are identified after even mild TBI and therefore may be sensitive to the underlying neural alterations associated with injury [5]. Thus, neurophysiological signals have the potential to aid in TBI diagnosis, prognosis, and treatment response monitoring [1,22,35].

In both humans and animals, TBI disrupts brain oscillations. More recently, studies have shown that electrical stimulation can restore oscillatory rhythms to improve cognitive outcomes following TBI. Generally, after injury, there is a decrease in power across frequency bands which returns to baseline after the acute injury response [1,36]. However, deficits in brain activity can persist chronically (>10 weeks) [36] and, thus, may represent a therapeutic window for clinical intervention. In patients with TBI, altered EEG activity can be seen years after injury [12,32,37,38]. Specifically, alterations of thalamo-cortical circuits are associated with persistent cognitive symptoms seen with TBI [5,21]. The dorsolateral prefrontal cortex, which is normally engaged during cognition, shows reduced power and EEG abnormalities after TBI [8]. Electrophysiological abnormalities occur across brain states (rest, sleep, etc.) and are consistent enough to distinguish between injured and non-injured patients and predict patient outcomes [32,38].

### 2.2. Neuromodulation to Restore Brain Activity

Modulating brain activity through electrical stimulation can restore rhythmic patterns of the brain disrupted by TBI [39]. To address the widespread neural impairments, stimulation would need to restore activity beyond the focal injury site. Functional MRI in humans and electrophysiological recordings in rodents show that brain stimulation emulates changes across networks [40].

Methods of neuromodulation vary in their level of invasiveness, continuous or transient application, and pairing with functional behavior or rest. Stimulation protocols can be open-loop (applied at/for a certain amount of time, not tied to a behavior or brain function), “on-demand” (triggered by a behavioral event), or closed-loop (triggered by brain feedback) [41,42]. High-frequency stimulation (>5 Hz) is thought to induce excitability and increase cerebral blood flow, whereas low-frequency stimulation (<1 Hz) typically induces inhibitory effects [43]. Factors such as the type of neuromodulation, duration of stimulation, and regularity required will determine the best target for neuromodulation.

Neurostimulation in the form of transcranial magnetic stimulation (TMS), transcranial direct current stimulation (tDCS), and deep brain stimulation (DBS), applied in preclinical models have successfully ameliorated behavioral symptoms of TBI, including motor, attention, memory, mood, and impulsivity impairments [35,44]. In preclinical studies, stimulation applied immediately to several weeks after trauma improved behavioral outcomes, whereas clinical applications of neuromodulation are not usually introduced until later stages of recovery [44]. Repeated TMS applied to the left dorsolateral prefrontal cortex has FDA approval to treat depression and therefore may also be useful in TBI patients exhibiting mood dysregulation, central pain, and blunted cognition or affect [43,45,46]. Importantly, compared to other interventions, growing evidence supports that TMS can have a long-lasting impact on neural circuits by inducing plasticity [47].

TMS has been used in rodent models with non-invasive coils, but the translational potential is obscured by stimulation site specificity and the use of restraints or anesthesia [48,49]. Instead, “TMS-like” approaches that use implanted electrodes opposed to topical coils, may be optimal when designing preclinical studies [35]. “TMS-like” protocols of 20 Hz stimulation trains applied repeatedly for 10 days to prelimbic cortex normalize depressive-like behaviors and reduce brain-derived neurotrophic factor levels in reward-related regions [50].

### 2.3. How Does Stimulation Restore Brain Function Following TBI?

Axonal shearing, swelling, chronic inflammation, and microglia activation perpetuate neural loss and tissue degeneration after trauma [22,51,52,53]. Stimulation provides neuroprotection, decreases apoptosis, reduces inflammation, directs cerebral blood flow, and induces neuroplasticity to improve cell health and restore neural circuits [35,43,44]. White matter regeneration (which may be particularly important for cognition) is achieved through optogenetic, pharmacogenetic, or indirect brain stimulation by encouraging glial cells to myelinate active axons [54,55]. In preclinical models of TBI, reversed cortical tissue loss, white matter regeneration, and increased intracellular signaling are directly related to behavioral improvements [56]. TDCS and TMS increase c-fos expression (a marker of neural activity) [57]. TDCS also increases brain-derived neurotrophic factor (BDNF) in stimulated cortical regions of injured brains, indicating a focal neuroplasticity response that is associated with cognitive improvements in spatial memory [58]. Similarly, TMS reduces glial fibrillary acidic protein (GFAP) expression, associated with astroglia cells and neural regeneration [2,59]. Improved neural health and signal transduction can re-establish physiological patterns. DBS reduces spontaneous neural firing and encourages activation of efferent pathways by increasing the release of GABA from interneurons to restore the excitatory/inhibitory balance [45,60].

## 3. A Potential Biomarker of Reward-Processing Deficits in TBI

The lack of mechanistic information regarding failure of cognitive networks precludes the utility of neuromodulation to treat chronic TBI deficits. Precision medicine approaches to treat TBI depend on finding neurophysiological biomarkers that reliably mark discrete cognitive functions like decision-making, planning, and memory.

### 3.1. Preclinical Models of Frontal TBI

Rodent models provide information about neurobiological mechanisms which are necessary to inform clinical interventions. Aside from notable differences in brain size, relative volume of brain regions, and presence/absence of cortical folding, the architecture and functional networks are largely preserved between humans and rodents [61]. Animal models of TBI offer control over injury specifications, ability to monitor injury progression, and invasive techniques that are not feasible in humans [62]. Limitations in these models include lack of continuity between protocols, challenge replicating the heterogeneous nature of TBI, broad timeframe of injury (acute, subacute, and chronic), and inability to model exact biomechanical parameters of injury [37,48]. Despite these limitations, rodent models of frontal TBI produce robust and predictable cognitive deficits [62]. Bilateral frontal controlled cortical impact (CCI) injury reliably produces impairments in impulse control and decision-making [63].

Animal models also enable large-scale recordings of in vivo brain activity. Multi-site electrodes simultaneously capture field potentials at different sites across, or between, networks. “Brain-wide” recordings measuring activity from up to 32 brain areas simultaneously, can be used to characterize networks operating at distinct oscillatory frequencies to support unique behaviors [64,65,66]. Although these electrodes record from within the brain, the information they provide (large-scale measure of brain oscillations) is like human EEG. The following section provides an example of how this technique can identify neurophysiological biomarkers and potential targets for neuromodulation.

### 3.2. Deficient Cortical Beta Oscillations Indicate Reward-Processing Issues After TBI

As shown in humans and animals, an injury to prefrontal cortex results in chronic executive function deficits, including attention, memory, and reward-guided decision making [13,67,68]. Damage causes lasting disruption to brain circuits through inflammation, gliosis, cell death, and alterations in microstructure, ultimately effecting neural communication. Changes in brain oscillations perpetuate disruptions throughout functional networks after cortical damage. Related to executive function deficits, fronto-parietal (attention) and fronto-striatal (decision-making) networks show reduced electrophysiological responses and connectivity consistent with slower reaction times and poor decision making [69,70].

Oscillatory activity, particularly at beta frequencies, deriving from the prefrontal cortex is important for top–down attention [71], executive control [72], sensorimotor integration [73], motor planning [74], and decision making [75]. Beta oscillations are correlated with the cortical microstructure (i.e., myelin density/integrity) and therefore susceptible to damage through TBI [76]. Even mild TBI leads to localized and widespread disruptions in beta oscillatory activity (measured by magnetoencephalography) [5]. Beta activity in cortico-striatal regions (prefrontal cortex, orbitofrontal cortex, anterior insula, ventral striatum, and basolateral amygdala) marks positive valence (i.e., rewarded outcomes) [77,78,79,80]. In humans, reward-evoked beta oscillations correlate with activation of (and coupling between) ventral striatum and medial prefrontal cortex, suggesting that beta frequency oscillations may coordinate the neural circuits involved in reward processing [80]. In rats, across multiple tasks of reward-guided decision-making, we consistently find increases in beta-frequency (15–30 Hz) oscillations during reward processing that reflects reward magnitude, reward probability, and subjective value [77].

Due to its superficial position in the brain, cortex is particularly susceptible to injury [10], giving rise to socially inappropriate behavior, poor impulse control, and trouble decision-making [21]. When cortical areas are damaged, their participating networks are also dysfunctional. As a network, cortico-striatal brain areas mediate adaptive reward-guided decision-making by creating action-outcome associations, controlling impulsive choices, and responding flexibly to changing conditions [81]. Bifrontal TBI caused by CCI decreases the ability of rats to detect reinforced outcomes, impairs behavioral flexibility, and increases impulsivity [63,82].

**Figure 2:**
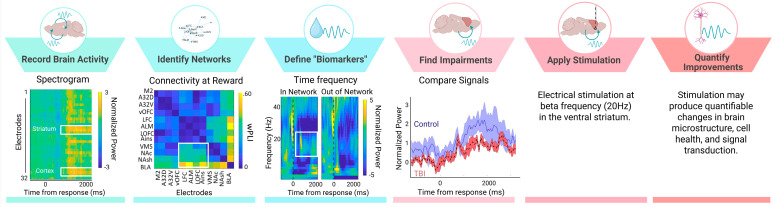
Reward-evoked beta oscillations as a putative neurophysiological marker. We apply large-scale beta oscillations to our theoretical framework to provide one example of how to identify neural activity associated with distinct behaviors to test neuromodulation targets in TBI. Neural signals were recorded across 32 CH in rodents performing behavioral tasks. We find areas of the cortico-striatal network that have heightened activity (spectrogram shows increased power at cortical and striatal electrodes) and connectivity (weighted phase lag index (wPLI) connectivity between pairs of electrodes) during reward. Evoked activity during reward occurs at beta frequencies (time frequency plot shows increased beta frequency activity in “within network” electrodes), thereby providing region (striatum) and frequency (beta) specificity needed for “biomarker” identification. In animals with TBI (red), beta oscillations during reward are blunted compared to controls (blue) (mean and SEM of evoked signal), and therefore may represent a logical target for neuromodulation. In the future, beta frequency (20 Hz) stimulation applied to the striatum should be tested in TBI animals and improvements in cell health, microstructure, and signal integrity should be quantified. Importantly, beta oscillations represent just one example of a neurophysiological signal that can be targeted to improve TBI symptoms. Our discussion includes examples of other neurophysiological signals. Created in BioRender. Koloski, M. (2025) https://BioRender.com/7xr6lr3 (accessed on 21 April 2025).

Consistent with these behavioral changes, we find that CCI-TBI also blunts reward-locked beta oscillations and cellular activity in lateral orbitofrontal cortex (measured by c-Fos staining) [82]. Beta activity in the orbitofrontal cortex does not discriminate between reward outcomes as efficiently following injury (effects observed up to 12 weeks after injury). Beta oscillations are correlated with structural deficits observed after TBI, including myelin density and morphology [76]. Local changes in beta power and deficits in functional connectivity following TBI has also been characterized in thalamo-cortical circuits [5,30,83]. Interestingly, we found that reductions in orbitofrontal activity were rescued by a behavioral intervention in which reinforced outcomes were cued, thus suggesting reward signals in the brain are malleable and can be altered to improve cognitive behavior [82]. Brain-based interventions may work similarly to increase beta oscillations during positive reward outcomes thereby reversing reward-related deficits from TBI. For these reasons, reward-evoked beta oscillations in the cortico-striatal network may represent a neurophysiological marker that can be targeted with electrical stimulation to improve cognitive symptoms of TBI. In accordance with the theoretical framework for neurophysiological marker identification (Figure 1), we have shown that large-scale recordings of brain activity can identify behaviorally relevant networks to define putative “biomarkers” with region and frequency specificity (Figure 2). We have found impairments of reward-evoked beta oscillations following TBI that may be remediated by electrical stimulation (Figure 2).

Although, we have not tested electrical stimulation on this putative beta signal in TBI animals, we have stimulated beta oscillations to influence decision making in healthy animals. Beta oscillations were modified in healthy rats performing a delayed discounting task, in which they chose between a small, immediate reward or a large, delayed reward [77]. In total, 20 Hz electrical stimulation was applied “on-demand”, triggered by an animal selecting the large, delayed reward outcome. Stimulation biased behavior toward the large reward choice, despite the temporal delay (2–10 s). Multiple cortico-striatal targets (including OFC) had this effect [77]. Although these results are limited due to lack of control/replication, they warrant testing beta-frequency modulation in an injured cohort.

While much of the current evidence regarding beta oscillations observed after TBI stems from our research, other studies have identified beta activity for reward processing and cognitive control across both humans and animal models [71,72,73,78,79]. Beta-evoked oscillations are seen during decision-making, particularly in situations with high cognitive demand [73]. In humans with mild TBI, deficits in frontal beta power (measured with magnetoencephalography) reflect thalamo-cortical network damage [5]. Beta-functional connectivity, compared with other frequencies, best predicted symptoms severity and mild TBI classification [5].

Contrary to these findings, some researchers have found evoked beta power following the omission of expected rewards [84]. Thus, although beta likely plays a role in reward processing, under what conditions it signals expectancy and reward outcome remain unclear. However, the role of beta oscillations following TBI warrants further investigation.

### 3.3. Other Potential Neurophysiological Biomarkers

Beta oscillations in the cortico-striatal network may represent a promising neurophysiological biomarker tied to a specific cognitive domain (reward valuation). Other neurophysiological markers pertaining to different cognitive domains may also be used to improve TBI outcomes with neuromodulation. For example, theta oscillations are suppressed after TBI and return over the course of recovery [30,83]. Theta oscillations are an appealing target to consider, as they are believed to modulate long-term potentiation underlying learning and memory [85,86]. Stimulating the medial septum (increasing theta oscillations) restored cognitive performance in rats with TBI [83,86,87]. Likewise, in rodent TBI models, gamma activity is decreased near the focal injury site and signal (and associated behavior consequences) is rescued with 40 Hz modulation (Blue LED photobiomodulation therapy) [88].

Another avenue that has been explored is modulating delta oscillations to change sleep dynamics (and thereby wakeful neural function) that are interrupted by TBI. Delta oscillations typically present in deep sleep are elevated during wakefulness after TBI [31]. Hypothalamic stimulation recovers delta oscillations to restore organized sleep after TBI [31].

## 4. Limitations and Challenges (In Translation and Beyond)

Despite promising preliminary findings, research on beta oscillations as a biomarker in TBI is still in its infancy. Generally, a role for beta oscillations in facilitating cognition (and reward evaluation) is accepted, but the disruption of beta signals in the case of TBI is less known. Beta oscillations are believed to facilitate top–down control of cognitive behaviors [89], and although these coincide with TBI sequalae, the involvement of beta oscillations explicitly still needs to be explored. Studies examining neurophysiological markers pertaining to cognitive deficits in TBI are sparse and often limited by methodological variability (injury models, severity, timeline, region of interest) across studies. Compared with deficits in motor function, cognitive networks are harder to precisely target and are often confounded by emotional/neuropsychiatric symptoms [90]. More preclinical research is needed to delineate the neurobiological substrates of cognitive behavior. Neurophysiological measures sometimes show weak or no associations with behavioral or functional outcomes. Barone et al., 2024, find that EEG alterations in mild TBI did not significantly correlate with clinical outcomes measures, underscoring the need to further refine which markers can best predict patient outcomes [27]. Although EEG is used clinically in an intensive-care setting to monitor seizure activity or drug effects, it is not common in outpatient settings [90]. Clinical assessment of EEG activity over a more chronic time course will help identify electrophysiological signatures related to individual pathologies.

Moreover, the success of neuromodulation to ameliorate cognitive symptoms of TBI is occluded by inconsistent protocols and translational barriers. Animal stimulation protocols are inconsistent in how they apply neuromodulation to TBI. Methodological factors like the type of stimulation (deep brain v. external), location, duration, and onset of stimulation relevant to TBI must be considered [44]. Further, factors like the use of anesthesia complicate methodological similarities and obscure translatability. While rodent models offer a high degree of experimental control, their translational value is often obscured by differences in anatomical division, cytoarchitecture, network structure, and behavior complexity. Moreover, clinical safety profiles and ideal stimulation parameters may be hard to gleam from rodent studies. The risk of seizures and adverse side effects with TMS or tDCS in TBI patients must be considered when generalizing across species [39,44,45]. Since patients with TBI present a high-risk population, the risk profile and safety guidelines of clinical neuromodulation must be carefully considered. Future work will need to identify homologous neural circuits, validate neurophysiological markers across species, and refine neuromodulation parameters for clinical application.

Finally, due to the heterogenous nature of TBI, multimodal treatments will likely be effective. Guided by biomarkers, stimulation may be paired with therapy or pharmacological treatment to address multiple symptoms. In humans, neuromodulation (tDCS) paired with cognitive training can enhance its success [90]. In some cases, enhancements in cognition were small and non-significant, further supporting the need to identify the networks to apply neuromodulation that will have maximal benefit when paired with cognitive rehabilitation [91]. Multimodal treatments create a challenge in ascertaining the exact mechanism, pathological target, and duration/frequency of treatment needed [44,90]. Without knowledge about which networks to target, it is possible that each treatment modality would target opposing networks and produce a null effect [90].

## 5. Conclusions

Even after identifying a reliable biomarker for intervention, important questions, like when in the disease progression to intervein and how long benefits persist, will need to be researched. Electrophysiology allows us to measure impairments which are undetectable by other methods, but using multi-modal diagnostic biomarkers (blood, structural, physiological, genetic) offers the most precision to treat individual pathologies by capturing the heterogeneous nature of TBI. The current lack of objective biomarkers for TBI poses a significant challenge for translational research. Future work should employ biomarkers to confirm that injuries induced in preclinical models are comparable to humans and that a proposed treatment would be clinically effective.

## References

[1] Wilde E.A., Wanner I.-B., Kenney K., Gill J., Stone J.R., Disner S., Schnakers C., Meyer R., Prager E.M., Haas M. (2022). A Framework to Advance Biomarker Development in the Diagnosis, Outcome Prediction, and Treatment of Traumatic Brain Injury. J. Neurotrauma.

[2] Ghaith H.S., Nawar A.A., Gabra M.D., Abdelrahman M.E., Nafady M.H., Bahbah E.I., Ebada M.A., Ashraf G.M., Negida A., Barreto G.E. (2022). A Literature Review of Traumatic Brain Injury Biomarkers. Mol. Neurobiol..

[3] Gutierre M.U., Telles J.P.M., Welling L.C., Rabelo N.N., Teixeira M.J., Figueiredo E.G. (2021). Biomarkers for Traumatic Brain Injury: A Short Review. Neurosurg. Rev..

[4] Bigler E.D. (2013). Neuroimaging Biomarkers in Mild Traumatic Brain Injury (mTBI). Neuropsychol. Rev..

[5] Zhang J., Safar K., Emami Z., Ibrahim G.M., Scratch S.E., Da Costa L., Dunkley B.T. (2020). Local and Large-Scale Beta Oscillatory Dysfunction in Males with Mild Traumatic Brain Injury. J. Neurophysiol..

[6] Freire M.A.M., Rocha G.S., Bittencourt L.O., Falcao D., Lima R.R., Cavalcanti J.R.L.P. (2023). Cellular and Molecular Pathophysiology of Traumatic Brain Injury: What Have We Learned So Far?. Biology.

[7] Ladak A.A., Enam S.A., Ibrahim M.T. (2019). A Review of the Molecular Mechanisms of Traumatic Brain Injury. World Neurosurg..

[8] Risbrough V.B., Vaughn M.N., Friend S.F. (2022). Role of Inflammation in Traumatic Brain Injury–Associated Risk for Neuropsychiatric Disorders: State of the Evidence and Where Do We Go From Here. Biol. Psychiatry.

[9] Chen Y.-H., Huang E.Y.-K., Kuo T.-T., Ma H.-I., Hoffer B.J., Tsui P.-F., Tsai J.-J., Chou Y.-C., Chiang Y.-H. (2015). Dopamine Release Impairment in Striatum after Different Levels of Cerebral Cortical Fluid Percussion Injury. Cell Transplant..

[10] Chou A., Morganti J.M., Rosi S. (2016). Frontal Lobe Contusion in Mice Chronically Impairs Prefrontal-Dependent Behavior. PLoS ONE.

[11] Polinder S., Cnossen M.C., Real R.G.L., Covic A., Gorbunova A., Voormolen D.C., Master C.L., Haagsma J.A., Diaz-Arrastia R., Von Steinbuechel N. (2018). A Multidimensional Approach to Post-Concussion Symptoms in Mild Traumatic Brain Injury. Front. Neurol..

[12] Gosselin N., Bottari C., Chen J.-K., Huntgeburth S.C., De Beaumont L., Petrides M., Cheung B., Ptito A. (2012). Evaluating the Cognitive Consequences of Mild Traumatic Brain Injury and Concussion by Using Electrophysiology. Neurosurg. Focus.

[13] Shaver T.K., Ozga J.E., Zhu B., Anderson K.G., Martens K.M., Vonder Haar C. (2019). Long-Term Deficits in Risky Decision-Making after Traumatic Brain Injury on a Rat Analog of the Iowa Gambling Task. Brain Res..

[14] Rabinowitz A.R., Levin H.S. (2014). Cognitive Sequelae of Traumatic Brain Injury. Psychiatr. Clin. N. Am..

[15] Rutherford W.H., Merrett J.D., McDonald J.R. (1979). Symptoms at One Year Following Concussion from Minor Head Injuries. Injury.

[16] Kundu B., Brock A.A., Englot D.J., Butson C.R., Rolston J.D. (2018). Deep Brain Stimulation for the Treatment of Disorders of Consciousness and Cognition in Traumatic Brain Injury Patients: A Review. Neurosurg. Focus.

[17] Kraus M.F., Susmaras T., Caughlin B.P., Walker C.J., Sweeney J.A., Little D.M. (2007). White Matter Integrity and Cognition in Chronic Traumatic Brain Injury: A Diffusion Tensor Imaging Study. Brain.

[18] Smith D.H., Meaney D.F. (2000). Axonal Damage in Traumatic Brain Injury. Neuroscientist.

[19] Stephens J.A., Williamson K.-N.C., Berryhill M.E. (2015). Cognitive Rehabilitation After Traumatic Brain Injury: A Reference for Occupational Therapists. OTJR Occup. Ther. J. Res..

[20] Yin B., Li D.-D., Huang H., Gu C.-H., Bai G.-H., Hu L.-X., Zhuang J.-F., Zhang M. (2019). Longitudinal Changes in Diffusion Tensor Imaging Following Mild Traumatic Brain Injury and Correlation With Outcome. Front. Neural Circuits.

[21] Dockree P.M., Robertson I.H. (2011). Electrophysiological Markers of Cognitive Deficits in Traumatic Brain Injury: A Review. Int. J. Psychophysiol..

[22] McNerney M.W., Gurkoff G.G., Beard C., Berryhill M.E. (2023). The Rehabilitation Potential of Neurostimulation for Mild Traumatic Brain Injury in Animal and Human Studies. Brain Sci..

[23] Corrigan F., Wee I.C., Collins-Praino L.E. (2023). Chronic Motor Performance Following Different Traumatic Brain Injury Severity-A Systematic Review. Front. Neurol..

[24] Hornby T.G., Reisman D.S., Ward I.G., Scheets P.L., Miller A., Haddad D., Fox E.J., Fritz N.E., Hawkins K., Henderson C.E. (2020). Clinical Practice Guideline to Improve Locomotor Function Following Chronic Stroke, Incomplete Spinal Cord Injury, and Brain Injury. J. Neurol. Phys. Ther. JNPT.

[25] Tani J., Wen Y.-T., Hu C.-J., Sung J.-Y. (2022). Current and Potential Pharmacologic Therapies for Traumatic Brain Injury. Pharmaceuticals.

[26] Stein M.B., Jain S., Giacino J.T., Levin H., Dikmen S., Nelson L.D., Vassar M.J., Okonkwo D.O., Diaz-Arrastia R., Robertson C.S. (2019). Risk of Posttraumatic Stress Disorder and Major Depression in Civilian Patients After Mild Traumatic Brain Injury: A TRACK-TBI Study. JAMA Psychiatry.

[27] Barone V., De Koning M.E., Van Der Horn H.J., Van Der Naalt J., Eertman-Meyer C.J., Van Putten M.J.A.M. (2024). Neurophysiological Signatures of Mild Traumatic Brain Injury in the Acute and Subacute Phase. Neurol. Sci..

[28] Buzsáki G., Anastassiou C.A., Koch C. (2012). The Origin of Extracellular Fields and Currents--EEG, ECoG, LFP and Spikes. Nat. Rev. Neurosci..

[29] Masimore B., Kakalios J., Redish A.D. (2004). Measuring Fundamental Frequencies in Local Field Potentials. J. Neurosci. Methods.

[30] Cavanagh J.F., Rieger R.E., Wilson J.K., Gill D., Fullerton L., Brandt E., Mayer A.R. (2020). Joint Analysis of Frontal Theta Synchrony and White Matter Following Mild Traumatic Brain Injury. Brain Imaging Behav..

[31] Dong X., Ye W., Tang Y., Wang J., Zhong L., Xiong J., Liu H., Lu G., Feng Z. (2021). Wakefulness-Promoting Effects of Lateral Hypothalamic Area-Deep Brain Stimulation in Traumatic Brain Injury-Induced Comatose Rats: Upregulation of A1-Adrenoceptor Subtypes and Downregulation of Gamma-Aminobutyric Acid β Receptor Expression Via the Orexins Pathway. World Neurosurg..

[32] Antón Álvarez X., Sampedro C., Pérez P., Laredo M., Couceiro V., Hernández Á., Figueroa J., Varela M., Arias D., Corzo L. (2003). Positive Effects of Cerebrolysin on Electroencephalogram Slowing, Cognition and Clinical Outcome in Patients with Postacute Traumatic Brain Injury: An Exploratory Study. Int. Clin. Psychopharmacol..

[33] Sherman M.T., Kanai R., Seth A.K., VanRullen R. (2016). Rhythmic Influence of Top–Down Perceptual Priors in the Phase of Prestimulus Occipital Alpha Oscillations. J. Cogn. Neurosci..

[34] Xiong Y., Fries P., Bastos A.M., Axmacher N. (2023). Which Rhythms Reflect Bottom-Up and Top-Down Processing?. Intracranial EEG.

[35] Surendrakumar S., Rabelo T.K., Campos A.C.P., Mollica A., Abrahao A., Lipsman N., Burke M.J., Hamani C. (2023). Neuromodulation Therapies in Pre-Clinical Models of Traumatic Brain Injury: Systematic Review and Translational Applications. J. Neurotrauma.

[36] Pevzner A., Izadi A., Lee D.J., Shahlaie K., Gurkoff G.G. (2016). Making Waves in the Brain: What Are Oscillations, and Why Modulating Them Makes Sense for Brain Injury. Front. Syst. Neurosci..

[37] Slobounov S., Sebastianelli W., Hallett M. (2012). Residual Brain Dysfunction Observed One Year Post-Mild Traumatic Brain Injury: Combined EEG and Balance Study. Clin. Neurophysiol..

[38] Thatcher R.W., Walker R.A., Gerson I., Geisler F.H. (1989). EEG Discriminant Analyses of Mild Head Trauma. Electroencephalogr. Clin. Neurophysiol..

[39] Demirtas-Tatlidede A., Vahabzadeh-Hagh A.M., Bernabeu M., Tormos J.M., Pascual-Leone A. (2012). Noninvasive Brain Stimulation in Traumatic Brain Injury. J. Head Trauma Rehabil..

[40] Tanaka Y., Matsuwaki T., Yamanouchi K., Nishihara M. (2013). Exacerbated Inflammatory Responses Related to Activated Microglia after Traumatic Brain Injury in Progranulin-Deficient Mice. Neuroscience.

[41] Bundy D.T., Nudo R.J. (2019). Preclinical Studies of Neuroplasticity Following Experimental Brain Injury: An Update. Stroke.

[42] Ghasemi P., Sahraee T., Mohammadi A. (2018). Closed- and Open-Loop Deep Brain Stimulation: Methods, Challenges, Current and Future Aspects. J. Biomed. Phys. Eng..

[43] Calderone A., Cardile D., Gangemi A., De Luca R., Quartarone A., Corallo F., Calabrò R.S. (2024). Traumatic Brain Injury and Neuromodulation Techniques in Rehabilitation: A Scoping Review. Biomedicines.

[44] Ziesel D., Nowakowska M., Scheruebel S., Kornmueller K., Schäfer U., Schindl R., Baumgartner C., Üçal M., Rienmüller T. (2023). Electrical Stimulation Methods and Protocols for the Treatment of Traumatic Brain Injury: A Critical Review of Preclinical Research. J. Neuroeng. Rehabil..

[45] Perera T., George M.S., Grammer G., Janicak P.G., Pascual-Leone A., Wirecki T.S. (2016). The Clinical TMS Society Consensus Review and Treatment Recommendations for TMS Therapy for Major Depressive Disorder. Brain Stimul..

[46] Pink A.E., Williams C., Alderman N., Stoffels M. (2021). The Use of Repetitive Transcranial Magnetic Stimulation (rTMS) Following Traumatic Brain Injury (TBI): A Scoping Review. Neuropsychol. Rehabil..

[47] Klomjai W., Katz R., Lackmy-Vallée A. (2015). Basic Principles of Transcranial Magnetic Stimulation (TMS) and Repetitive TMS (rTMS). Ann. Phys. Rehabil. Med..

[48] Vahabzadeh-Hagh A.M., Muller P.A., Gersner R., Zangen A., Rotenberg A. (2012). Translational Neuromodulation: Approximating Human Transcranial Magnetic Stimulation Protocols in Rats. Neuromodulation Technol. Neural Interface.

[49] Boonzaier J., Petrov P.I., Otte W.M., Smirnov N., Neggers S.F.W., Dijkhuizen R.M. (2020). Design and Evaluation of a Rodent-Specific Transcranial Magnetic Stimulation Coil: An In Silico and In Vivo Validation Study. Neuromodulation.

[50] Moshe H., Gal R., Barnea-Ygael N., Gulevsky T., Alyagon U., Zangen A. (2016). Prelimbic Stimulation Ameliorates Depressive-Like Behaviors and Increases Regional BDNF Expression in a Novel Drug-Resistant Animal Model of Depression. Brain Stimul..

[51] Collins-Praino L.E. (2022). Traumatic Axonal Injury as a Key Driver of the Relationship between Traumatic Brain Injury, Cognitive Dysfunction, and Dementia. Cellular, Molecular, Physiological, and Behavioral Aspects of Traumatic Brain Injury.

[52] Smith D.H., Kochanek P.M., Rosi S., Meyer R., Ferland-Beckham C., Prager E.M., Ahlers S.T., Crawford F. (2021). Roadmap for Advancing Pre-Clinical Science in Traumatic Brain Injury. J. Neurotrauma.

[53] Xu H., Wang Z., Li J., Wu H., Peng Y., Fan L., Chen J., Gu C., Yan F., Wang L. (2017). The Polarization States of Microglia in TBI: A New Paradigm for Pharmacological Intervention. Neural Plast..

[54] Johnson V.E., Stewart J.E., Begbie F.D., Trojanowski J.Q., Smith D.H., Stewart W. (2013). Inflammation and White Matter Degeneration Persist for Years after a Single Traumatic Brain Injury. Brain.

[55] Maas A.I.R., Menon D.K., Manley G.T., Abrams M., Åkerlund C., Andelic N., Aries M., Bashford T., Bell M.J., Bodien Y.G. (2022). Traumatic Brain Injury: Progress and Challenges in Prevention, Clinical Care, and Research. Lancet Neurol..

[56] Carballosa Gonzalez M.M., Blaya M.O., Alonso O.F., Bramlett H.M., Hentall I.D. (2013). Midbrain Raphe Stimulation Improves Behavioral and Anatomical Recovery from Fluid-Percussion Brain Injury. J. Neurotrauma.

[57] Yoon Y.-S., Cho K.H., Kim E.-S., Lee M.-S., Lee K.J. (2015). Effect of Epidural Electrical Stimulation and Repetitive Transcranial Magnetic Stimulation in Rats With Diffuse Traumatic Brain Injury. Ann. Rehabil. Med..

[58] Yu K.P., Yoon Y.-S., Lee J.G., Oh J.S., Lee J.-S., Seog T., Lee H.-Y. (2018). Effects of Electric Cortical Stimulation (ECS) and Transcranial Direct Current Stimulation (tDCS) on Rats With a Traumatic Brain Injury. Ann. Rehabil. Med..

[59] Sekar S., Zhang Y., Miranzadeh Mahabadi H., Parvizi A., Taghibiglou C. (2019). Low-Field Magnetic Stimulation Restores Cognitive and Motor Functions in the Mouse Model of Repeated Traumatic Brain Injury: Role of Cellular Prion Protein. J. Neurotrauma.

[60] Chiken S., Nambu A. (2016). Mechanism of Deep Brain Stimulation: Inhibition, Excitation, or Disruption?. Neuroscientist.

[61] Xu N., LaGrow T.J., Anumba N., Lee A., Zhang X., Yousefi B., Bassil Y., Clavijo G.P., Khalilzad Sharghi V., Maltbie E. (2022). Functional Connectivity of the Brain Across Rodents and Humans. Front. Neurosci..

[62] Ma X., Aravind A., Pfister B.J., Chandra N., Haorah J. (2019). Animal Models of Traumatic Brain Injury and Assessment of Injury Severity. Mol. Neurobiol..

[63] Vonder Haar C., Lam F.C.W., Adams W.K., Riparip L.-K., Kaur S., Muthukrishna M., Rosi S., Winstanley C.A. (2016). Frontal Traumatic Brain Injury in Rats Causes Long-Lasting Impairments in Impulse Control That Are Differentially Sensitive to Pharmacotherapeutics and Associated with Chronic Neuroinflammation. ACS Chem. Neurosci..

[64] Francoeur M.J., Tang T., Fakhraei L., Wu X., Hulyalkar S., Cramer J., Buscher N., Ramanathan D.R. (2021). Chronic, Multi-Site Recordings Supported by Two Low-Cost, Stationary Probe Designs Optimized to Capture Either Single Unit or Local Field Potential Activity in Behaving Rats. Front. Psychiatry.

[65] Fakhraei L., Francoeur M., Balasubramani P., Tang T., Hulyalkar S., Buscher N., Claros C., Terry A., Gupta A., Xiong H. (2021). Mapping Large-Scale Networks Associated with Action, Behavioral Inhibition and Impulsivity. eNeuro.

[66] Fakhraei L., Francoeur M., Balasubramani P.P., Tang T., Hulyalkar S., Buscher N., Mishra J., Ramanathan D.S. (2021). Electrophysiological Correlates of Rodent Default-Mode Network Suppression Revealed by Large-Scale Local Field Potential Recordings. Cereb. Cortex Commun..

[67] Paterno R., Folweiler K.A., Cohen A.S. (2017). Pathophysiology and Treatment of Memory Dysfunction After Traumatic Brain Injury. Curr. Neurol. Neurosci. Rep..

[68] VanSolkema M., McCann C., Barker-Collo S., Foster A. (2020). Attention and Communication Following TBI: Making the Connection through a Meta-Narrative Systematic Review. Neuropsychol. Rev..

[69] Kim N., Jamison K., Jaywant A., Garetti J., Blunt E., RoyChoudhury A., Butler T., Dams-O’Connor K., Khedr S., Chen C.-C. (2023). Comparisons of Electrophysiological Markers of Impaired Executive Attention after Traumatic Brain Injury and in Healthy Aging. NeuroImage.

[70] De Simoni S., Jenkins P.O., Bourke N.J., Fleminger J.J., Hellyer P.J., Jolly A.E., Patel M.C., Cole J.H., Leech R., Sharp D.J. (2018). Altered Caudate Connectivity Is Associated with Executive Dysfunction after Traumatic Brain Injury. Brain.

[71] Schmidt R., Herrojo Ruiz M., Kilavik B.E., Lundqvist M., Starr P.A., Aron A.R. (2019). Beta Oscillations in Working Memory, Executive Control of Movement and Thought, and Sensorimotor Function. J. Neurosci..

[72] Spitzer B., Haegens S. (2017). Beyond the Status Quo: A Role for Beta Oscillations in Endogenous Content (Re)Activation. eNeuro.

[73] Dunkley B.T., Freeman T.C.A., Muthukumaraswamy S.D., Singh K.D. (2013). Cortical Oscillatory Changes in Human Middle Temporal Cortex Underlying Smooth Pursuit Eye Movements. Hum. Brain Mapp..

[74] Little S., Bonaiuto J., Barnes G., Bestmann S. (2019). Human Motor Cortical Beta Bursts Relate to Movement Planning and Response Errors. PLOS Biol..

[75] Hoy C.W., De Hemptinne C., Wang S.S., Harmer C.J., Apps M.A.J., Husain M., Starr P.A., Little S. (2024). Beta and Theta Oscillations Track Effort and Previous Reward in Human Basal Ganglia and Prefrontal Cortex during Decision Making. Proc. Natl. Acad. Sci. USA.

[76] Hunt A.W., Mah K., Reed N., Engel L., Keightley M. (2016). Oculomotor-Based Vision Assessment in Mild Traumatic Brain Injury: A Systematic Review. J. Head Trauma Rehabil..

[77] Koloski M.F., Hulyalkar S., Barnes S.A., Mishra J., Ramanathan D.S. (2024). Cortico-Striatal Beta Oscillations as a Reward-Related Signal. Cogn. Affect. Behav. Neurosci..

[78] HajiHosseini A., Holroyd C.B. (2015). Sensitivity of Frontal Beta Oscillations to Reward Valence but Not Probability. Neurosci. Lett..

[79] Marco-Pallarés J., Münte T.F., Rodríguez-Fornells A. (2015). The Role of High-Frequency Oscillatory Activity in Reward Processing and Learning. Neurosci. Biobehav. Rev..

[80] Mas-Herrero E., Ripollés P., HajiHosseini A., Rodríguez-Fornells A., Marco-Pallarés J. (2015). Beta Oscillations and Reward Processing: Coupling Oscillatory Activity and Hemodynamic Responses. NeuroImage.

[81] D’Cruz A.-M., Ragozzino M.E., Mosconi M.W., Pavuluri M.N., Sweeney J.A. (2011). Human Reversal Learning under Conditions of Certain versus Uncertain Outcomes. NeuroImage.

[82] Koloski M.F., O’Hearn C.M., Frankot M., Giesler L.P., Ramanathan D.S., Vonder Haar C. (2024). Behavioral Interventions Can Improve Brain Injury-Induced Deficits in Behavioral Flexibility and Impulsivity Linked to Impaired Reward-Feedback Beta Oscillations. J. Neurotrauma.

[83] Lee D.J., Gurkoff G.G., Izadi A., Seidl S.E., Echeverri A., Melnik M., Berman R.F., Ekstrom A.D., Muizelaar J.P., Lyeth B.G. (2015). Septohippocampal Neuromodulation Improves Cognition after Traumatic Brain Injury. J. Neurotrauma.

[84] Yaple Z., Martinez-Saito M., Novikov N., Altukhov D., Shestakova A., Klucharev V. (2018). Power of Feedback-Induced Beta Oscillations Reflect Omission of Rewards: Evidence From an EEG Gambling Study. Front. Neurosci..

[85] Hyman J.M., Wyble B.P., Goyal V., Rossi C.A., Hasselmo M.E. (2003). Stimulation in Hippocampal Region CA1 in Behaving Rats Yields Long-Term Potentiation When Delivered to the Peak of Theta and Long-Term Depression When Delivered to the Trough. J. Neurosci..

[86] Lee D.J., Gurkoff G.G., Izadi A., Berman R.F., Ekstrom A.D., Muizelaar J.P., Lyeth B.G., Shahlaie K. (2013). Medial Septal Nucleus Theta Frequency Deep Brain Stimulation Improves Spatial Working Memory after Traumatic Brain Injury. J. Neurotrauma.

[87] Lee D.J., Izadi A., Melnik M., Seidl S., Echeverri A., Shahlaie K., Gurkoff G.G. (2017). Stimulation of the Medial Septum Improves Performance in Spatial Learning Following Pilocarpine-Induced Status Epilepticus. Epilepsy Res..

[88] Yang X., Li X., Yuan Y., Sun T., Yang J., Deng B., Yu H., Gao A., Guan J. (2022). 40 Hz Blue LED Relieves the Gamma Oscillations Changes Caused by Traumatic Brain Injury in Rat. Front. Neurol..

[89] Lundqvist M., Miller E.K., Nordmark J., Liljefors J., Herman P. (2024). Beta: Bursts of Cognition. Trends Cogn. Sci..

[90] Zaninotto A.L., El-Hagrassy M.M., Green J.R., Babo M., Paglioni V.M., Benute G.G., Paiva W.S. (2019). Transcranial Direct Current Stimulation (tDCS) Effects on Traumatic Brain Injury (TBI) Recovery: A Systematic Review. Dement. Neuropsychol..

[91] Leśniak M., Polanowska K., Seniów J., Członkowska A. (2014). Effects of Repeated Anodal tDCS Coupled With Cognitive Training for Patients With Severe Traumatic Brain Injury: A Pilot Randomized Controlled Trial. J. Head Trauma Rehabil..

